# Multi-centre phase II clinical trial of yttrium-90 resin microspheres alone in unresectable, chemotherapy refractory colorectal liver metastases

**DOI:** 10.1038/sj.bjc.6605770

**Published:** 2010-07-13

**Authors:** M Cosimelli, R Golfieri, P P Cagol, L Carpanese, R Sciuto, C L Maini, R Mancini, I Sperduti, G Pizzi, M G Diodoro, M Perrone, E Giampalma, B Angelelli, F Fiore, S Lastoria, S Bacchetti, D Gasperini, O Geatti, F Izzo

**Affiliations:** 1 Regina Elena National Cancer Institute, Via Elio Chianesi, 53, 00144 Rome, Italy; 2 S Orsola-Malpighi University Hospital, Bologna, Italy; 3 University of Udine, Udine, Italy; 4 Fondazione Pascale Cancer Institute of Naples, Naples, Italy

**Keywords:** radioembolisation, selective internal radiation therapy, SIRT, chemorefractory, colorectal cancer, metastases

## Abstract

**Background:**

This multi-centre phase II clinical trial is the first prospective evaluation of radioembolisation of patients with colorectal liver metastases (mCRC) who failed previous oxaliplatin- and irinotecan-based systemic chemotherapy regimens.

**Methods:**

Eligible patients had adequate hepatic, haemopoietic and renal function, and an absence of major hepatic vascular anomalies and hepato-pulmonary shunting. Gastroduodenal and right gastric arteries were embolised before hepatic arterial administration of yttrium-90 resin microspheres (median activity, 1.7 GBq; range, 0.9–2.2).

**Results:**

Of 50 eligible patients, 38 (76%) had received ⩾4 lines of chemotherapy. Most presented with synchronous disease (72%), >4 hepatic metastases (58%), 25–50% replacement of total liver volume (60%) and bilateral spread (70%). Early and intermediate (>48 h) WHO G1–2 adverse events (mostly fever and pain) were observed in 16 and 22% of patients respectively. Two died due to renal failure at 40 days or liver failure at 60 days respectively. By intention-to-treat analysis using Response Evaluation Criteria in Solid Tumours, 1 patient (2%) had a complete response, 11 (22%) partial response, 12 (24%) stable disease, 22 (44%) progressive disease; 4 (8%) were non-evaluable. Median overall survival was 12.6 months (95% CI, 7.0–18.3); 2-year survival was 19.6%.

**Conclusion:**

Radioembolisation produced meaningful response and disease stabilisation in patients with advanced, unresectable and chemorefractory mCRC.

Colorectal cancer (CRC) is a leading cause of cancer-related death (Bipat *et al*, 2007). During 2006, there were 412 900 new cases of primary CRC diagnosed in Europe, and 207 500 deaths were reported (Ferlay *et al*, 2006). The liver is the most common visceral site of CRC metastasis (mCRC) and recurrence, and consequently the main cause of morbidity and mortality among this patient group (McMillan and McArdle, 2007). Approximately 15–25% of people diagnosed with CRC are affected by synchronous hepatic metastases whereas a further 15–20% of patients will develop metachronous liver metastases within 3 years following resection of the primary tumour (Manfredi *et al*, 2006). If untreated, the median survival of patients with mCRC is 6–8 months (Poston, 2004). Less than 25% of patients are surgical candidates, due to the position, size or number of the liver lesions (Khatri *et al*, 2007). Nevertheless, some 65–72% of patients will experience a recurrence of their hepatic tumours within 3 years following resection, with or without peri-operative chemotherapy (Nordlinger *et al*, 2008). For the majority of unresectable patients, the use of modern polychemotherapy regimens in combination with targeted agents has substantially extended median survival time, which is presently at 20–24 months (Hurwitz *et al*, 2004; Falcone *et al*, 2007). However, the majority of patients with mCRC will progress unless surgically resected. There remains a high medical need for effective treatments for patients with CRC liver metastases who have failed conventional chemotherapy regimens.

Radioembolisation delivers targeted radiation therapy to inoperable primary and secondary (i.e., metastatic) hepatic malignancies. It may be used simultaneously with chemotherapy to improve treatment-related response and prolong time to disease progression and survival compared with chemotherapy alone (Gray *et al*, 2001; van Hazel *et al*, 2004), or as a monotherapy either during a chemotherapy treatment hiatus or in patients with chemotherapy refractory disease, where it is emerging as an important and useful treatment option (Kennedy *et al*, 2006; Jakobs *et al*, 2008). Radioembolisation uses yttrium-90 (^90^Y), which is permanently bound to biocompatible, non-biodegradable microspheres. Yttrium-90 is a pure-*β* emitter that decays to stable zirconium-90 with an average energy of 0.94 MeV and a half-life of 2.67 days (64.2 h). One GBq (27 mCi) of ^90^Y delivers a high (total dose of 50 Gy kg^−1^) but very localised dose of *β*-radiation with a mean tissue penetration of 2.5 mm and a maximum range of 11 mm (Kennedy *et al*, 2004). The ^90^Y microspheres are delivered through a temporary transfemoral catheter advanced under fluoroscopic guidance into the hepatic artery branches that supply the metastatic tumours. The microspheres preferentially lodge in the neovascular rim of the tumour(s) and deliver tumouricidal doses of radiation (Kennedy *et al*, 2004).

The aim of the study was to evaluate the efficacy (clinical and radiographic responses, time to response, overall survival and progression-free survival) and tolerability of a single hepatic intra-arterial injection of ^90^Y resin microspheres (SIR-Spheres; Sirtex Medical Limited, Lane Cove, Australia) in patients with unresectable, chemotherapy refractory CRC liver metastases as the sole or dominant site of disease.

## Patients and methods

### Patients

This was a prospective, multi-centre phase II trial in patients with unresectable, histologically proven CRC adenocarcinoma liver metastases and limited extra-hepatic disease (⩽3 nodules in the same extra-hepatic organ each <3 mm as assessed by 64-slice computed tomography (CT)). Patients with liver disease progression following standard systemic chemotherapy (including FOLFOX and FOLFIRI regimens) were recruited following multidisciplinary review from four centres in Italy between May 2005 and August 2007. All patients were between 18 and 75 years of age, had liver metastases (measurable by Response Evaluation Criteria in Solid Tumours, RECIST), adequate renal function (creatinine<1.5 × normal values or creatinine clearance >50 ml min^−1^), haemopoietic function (leucocytes >1500 per mm^3^; platelet count >100 000 per mm^3^), WHO or ECOG performance status ⩽2 and were able to give informed consent. In addition, eligible patients were required to have (1) sufficient liver function for radioembolisation (defined as absence of ascites or synthetic liver dysfunction, together with total bilirubin<1.5 mg per 100 ml (<25.65 *μ*mol l^−1^), and AST, ALT and alkaline phosphatase each<4 × upper limit of normal); (2) hepatic arterial anatomy that would enable safe delivery of microspheres to the liver only; (3) liver to lung shunting of <20% on a pre-treatment technetium-99m-labelled macro-aggregated albumin (^99m^Tc-MAA) nuclear scan and (4) a patent main portal vein. Patients were excluded if they were pregnant, had evidence of local recurrence of primary disease, inflammatory gastrointestinal disease or had received previous treatment with hepatic arterial chemotherapy or external beam radiotherapy to the liver.

### Treatment

In all potentially eligible patients, CT was performed to define the location and extent of hepatic tumour involvement, followed by biopsy of one of the metastases, which was subject to a separate process of informed consent. Before radioembolisation, meticulous coeliac and superior mesenteric angiography was undertaken to map the hepatic arterial tree, identify arterial feeders to the gastrointestinal tract, and coil embolise the gastroduodenal and right gastric arteries and any other gastrointestinal tract feeders in the majority (93.8%) of patients. Once the hepatic arterial blood supply had been isolated, the ^99m^Tc-MAA injection was delivered into the proper hepatic artery in 75.0% of patients. In patients with aberrant hepatic arteries, two or more separate injections were performed in six patients and embolisation of vessels to redistribute blood flow was performed in three. Patients were then placed under a *γ*-camera to determine the extent of hepato-pulmonary shunting. The patient's body surface area (BSA) was determined using standard height/weight tables, with the activity of ^90^Y resin microspheres to be implanted calculated using the formula: 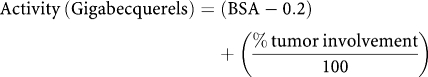


Among patients with disease limited to a single lobe, we used the lesion and lobe volume to calculate the percentage tumour involvement using the BSA formula at one centre in four patients; other treatment centres used whole-liver volumes. The activity to be implanted into the liver was reduced by 20 or 40% if the pre-treatment ^99m^Tc-MAA study showed hepato-pulmonary shunting of 11–15% or 16–20%. At 1–2 weeks after the initial mapping angiogram, a second transfemoral hepatic arterial catheterisation was performed during which ^90^Y resin microspheres were administered into the proper hepatic artery under fluoroscopic guidance as a single whole-liver procedure. All patients were admitted on the day of the procedure and discharged 1 or 2 days later.

### Assessments

Haematological, liver function and blood biochemistry tests and physical examination were performed pre-treatment and on days 1, 8 and 30 and then at 6-week intervals after treatment. Radiation-induced pneumonitis was evaluated by X-ray on day 8 and then subsequently by chest–abdomen–pelvis CT scans.

Patients were assessed at 6-week intervals by chest–abdomen–pelvis CT scan for tumour response. A confirmatory CT scan was performed not less than 6 weeks later in the event that a partial or complete response (CR) or stable disease (SD) was detected. Efficacy outcome rates were assessed by RECIST. Disease progression was monitored at 6- or 12-week intervals.

The nature and severity of all adverse events were assessed and recorded from the time of the initiation of protocol treatment up to 3 months after treatment. At the time of their occurrence, adverse events were attributed as being definitely, probably, possibly, unlikely or not related to radioembolisation. At the end of the study, a central review of adverse events and imaging responses was conducted.

A psychological evaluation was carried out using a battery of tests before radioembolisation and at 6 weeks after treatment. Patients were assessed using both cancer- and disease-specific questionnaires for quality of life evaluation (EORTC QLQ C30, EORTC QLQ CR38, EORTC QLQ LMC-21), an anxiety and depression evaluation scale (HADs), and a patient satisfaction questionnaire (EORTC QLQ SAT-32).

The primary end point of this trial was the objective tumour response rate (ORR) after a single intra-arterial injection of ^90^Y resin microspheres. Secondary end points included tolerability, quality of life, duration of response, time to disease progression and overall survival assessed from the time of initiation of therapy. The trial was performed according to the Declaration of Helsinki Principles as well as the European Medicine Agency Guidance on Good Clinical Practice (CPMP/ICH/135/95; 17 July 1996). The study protocol was approved by the institutional review boards and ethics committee.

### Statistical design

This phase II trial was planned as a single-stage design as described by A’Hern (2001). The planned sample size of 48 patients was considered sufficient to give an 80% probability of rejecting a baseline response rate of 15% with an exact 5% one-sided significance test when the true response rate was 30%. The treatment was rejected if <12 responses were observed. Efficacy and safety were evaluated according to an intention-to-treat analysis. The association between variables was tested by the Pearson's *χ*^2^-test or the Fisher's exact test. Overall survival and progression-free survival were calculated by the Kaplan–Meier product-limit method from the date of radioembolisation until progression of disease or death from any cause or from malignant disease. If a patient had not progressed or died, survival and progression were censored at the time of their last visit. The log-rank test assessed differences between subgroups. Significance was defined at the *P*<0.05 level. SPSS 13.0 statistical software (IBM, Chicago, IL, USA) was used for analysis.

## Results

A total of 52 patients were enrolled and 50 patients (41 with colon and 9 with rectal primary sites) were included in the final analyses. Two patients were excluded due to excessive extra-hepatic disease. The median follow-up period was 11 months (range, 2–29); two patients were lost to follow-up. Baseline characteristics are outlined in Table 1. Most patients presented with synchronous (stage IV) disease (72%), >4 hepatic metastases (58%) (median size, 50 mm; range, 8–120), tumour involving 25–50% of the liver tissue (60%) and bilateral spread (70%). Eleven patients (22%) with limited extra-hepatic disease entered the trial (all with lung metastases and one with retroperitoneal lymph node metastases). Twelve patients (24%) had undergone previous hepatic resection of CRC metastases. All 50 patients (100%) had received >3 lines of systemic chemotherapy including at least one oxaliplatin- and one irinotecan-containing regimen; 38 (76%) received ⩾4 lines of previous chemotherapy. Eleven patients (22%) had been treated with bevacizumab and five (10%) with cetuximab. All patients, except one, had stopped oxaliplatin- or irinotecan-containing regimens due to disease progression.

The median implanted activity of ^90^Y resin microspheres was 1.7 GBq (range, 0.9–2.2). One patient required a 20% reduction in implanted activity due to lung shunting exceeding 10% and in another patient, the implanted activity was limited by stasis. In eight patients from a single centre, the implanted activity was reduced below the calculated activity due to concerns over the extent of pre-treatment and/or potential subsequent toxicity. The median interval between diagnosis of mCRC and radioembolisation was 17 months (range, 6–71). Repeat radioembolisation was performed in one patient at 1 month to improve the treatment response and in a further two patients to treat progression at 5 and 10 months respectively. Fourteen patients with progressive disease (PD) had further systemic chemotherapy after radioembolisation.

### Treatment response

A total of 46 patients were evaluable for response by RECIST; confirmatory scans on four patients were not available, but these patients were included in the intention-to-treat analysis for efficacy. Median time between the procedure and the maximum treatment response recorded by RECIST was 6 weeks (range, 6–12). The confirmed ORR (partial or CR) was 24.0% (95% confidence interval (CI), 12.2–35.8%) by RECIST, which met the pre-determined criteria for significance (*P*=0.05). One patient (2.0%) had a CR, 11 (22.0%) a partial response (PR), 12 (24.0%) SD and 22 (44.0%) PD. Among responders, the median maximum diameter of nodules diminished from 50 mm (range, 25–64) to 35 mm. Treatment response was independent of performance status (0 *vs* 1–3, *P*=0.26), number of metastases (⩽4 *vs* >4, *P*=0.19), metastases size (⩽50 mm *vs* >50 mm, *P*=0.69), liver involvement (<25% *vs* 25–50%, *P*=0.74), previous anti-angiogenic agents (bevacizumab *vs* none, *P*=0.52) or previous resection (*P*=0.87). There was no significant difference between responders and non-responders in the median (±s.d.) total activity administered (1.6±0.28 GBq *vs* 1.65±0.3 GBq, *P*=0.33) or median total liver volume treated (1444.22±309.06 *vs* 1753.82±444.82, *P*=0.31).

Of the 14 patients who subsequently received chemotherapy for disease progression at 3 months (*n*=1), 4 months (*n*=2) and beyond (*n*=11) after radioembolisation, 3 had a treatment response (CR or PR).

**Figure 1:**
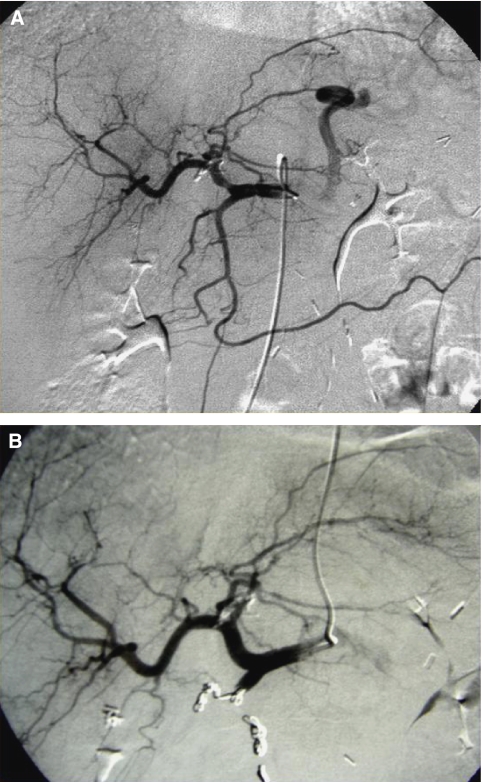
Preliminary angiographic evaluation (**A**) and subsequent administration of ^90^Y resin microspheres (**B**) after embolisation of gastroduodenal artery and branches in 64-year-old man with CRC liver metastases. Small diffuse hypervascular areas are observed throughout the liver parenchyma, confirming infiltrative malignant disease.

**Figure 2:**
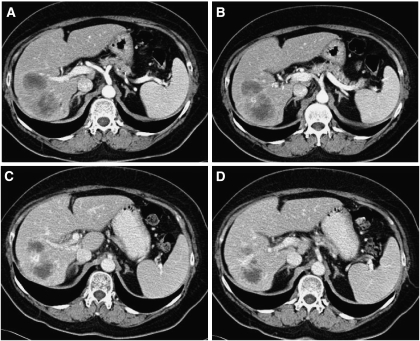
Contrast-enhanced CT scans of patient in Figure 1, showing pre-radioembolisation hypoattenuating lesions (**A/B**); 3-month post-radioembolisation decreased lesion size, decreased intra-lesional vascular enhancement, increased intra-lesional necrosis, thin peripheral enhancement (**C/D**) and reduced compression/narrowing of large posterior right portal vein branch (**D**).

Figures 1 and 2 depict the treatment of a 64-year-old patient who received radioembolisation after progressing despite treatment with four lines of chemotherapy for multiple bilobar CRC metastases. Figure 1 shows the preliminary angiographic evaluation and subsequent evaluation following administration of ^90^Y resin microspheres to the whole liver through the proper hepatic artery. The CT imaging after treatment (Figure 2) shows evidence of decreasing lesion size, intra-lesional necrosis and an improvement in the visualisation of the main right portal vein branch (which is an indirect sign of vein patency). The patient survived 1 year with no further lines of treatment.

**Figure 3:**
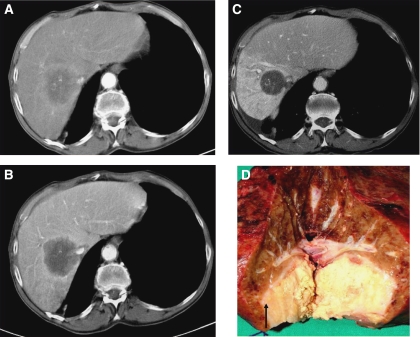
Contrast-enhanced pre-radioembolisation arterial (**A**) and portal-venous-phase (**B**) CT scans showing large CRC liver metastasis. 6-month post-radioembolisation (**C**) including significant attenuation, sharp margins and thin peripheral enhancement compatible with complete lesion necrosis; confirmed by post-resection evaluation (**D**), showing fibrotic capsule (arrow).

Two patients (4.0%) experienced sufficient reduction in the volume of their liver metastases to enable potentially curative resection of ⩾3 segments. Figure 3 shows the tumour response for one of these resected patients after ^90^Y resin microspheres were administered through the proper hepatic artery. This patient was initially considered unresectable due to vascular infiltration at the confluence of the right hepatic vein and inferior vena cava.

### Progression

Of the 22 patients who initially progressed, 3.6% of patients had intra-hepatic progression, 28.6% intra- and extra-hepatic progression and 14.3% extra-hepatic progression only. For these patients, time to intra-hepatic progression was 2.8 months, 3.2 months for intra- and extra-hepatic progression, and 4.5 months for extra-hepatic only.

### Survival

**Figure 4:**
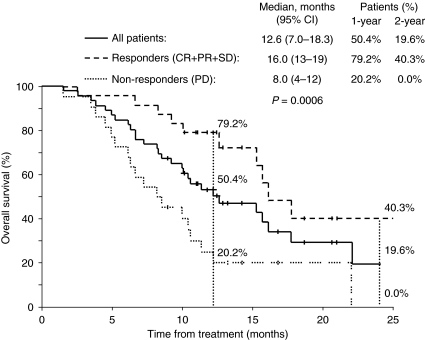
Kaplan–Meier plots of overall survival following radioembolisation with ^90^Y resin microspheres in unresectable, chemotherapy refractory CRC liver metastases in all patients (*n*=50); and among responders (CR+PR+SD; *n*=24) and non-responders (PD; *n*=22).

The median time to progression and progression-free survival was 3.7 months (95% CI, 2.6–4.9). Median overall survival was 12.6 months (95% CI, 7.0–18.3) with 1- and 2-year survival rates of 50.4 and 19.6% respectively (Figure 4). The median survival from first diagnosis of CRC liver metastases and death or the end of this study was 31 months (95% CI, 29–34).

There was a significant difference in survival between patients showing a response to radioembolisation (CR+PR+SD, *n*=24) and those who did not respond (PD, *n*=22) (16 *vs* 8 months, *P*=0.0006; Figure 4). Survival among responders and non-responders was 79.2 and 20.2% at 1 year and 40.3 and 0% at 2 years respectively.

### Adverse events

One patient died 40 days after treatment from acute renal failure and another responding patient died 60 days after treatment due to liver failure. Liver and kidney function tests in both patients were normal before treatment. Both deaths were classified as possibly related to treatment. All other adverse events, whether early (within the first 48 h), intermediate (within the first month) or late (2–3 months after treatment) were mild or moderate in nature (WHO grade 1/2 adverse events) (Table 2).

### Quality of life

Quality of life, as measured by cancer- and site-specific questionnaires (EORTC QLQ C30 and EORTC QLQ CR38) in 14 patients at 6 weeks, was not adversely affected by radioembolisation. Patients were satisfied with healthcare providers’ interpersonal and technical skills as well as the information provided on the treatment. Patients showed a good compliance to physicians’ advice (mean score of 8 on a visual analogue scale where ‘0’ is a low and ‘10’ is a high compliance score). The mean HADs scored ‘8’ for anxiety and ‘9’ for depression, indicating borderline pre-treatment levels of anxiety and depression. Six weeks after radioembolisation, patients’ anxiety levels were significantly reduced (*P*<0.01); with no significant change in depression score.

## Discussion

This multi-centre phase II clinical trial is the first prospective evaluation of radioembolisation using ^90^Y resin microspheres for inoperable CRC liver metastases in patients who had progressed on previous oxaliplatin- and irinotecan-based chemotherapy regimens. In heavily pre-treated patients refractory to the standard modern chemotherapeutic options, radioembolisation with ^90^Y resin microspheres produced meaningful median response rates with a low toxicity profile and little or no detrimental effect on health-related quality of life surveys. Disease control, including SD, following radioembolisation occurred in nearly half of treated patients and was associated with a longer survival compared with non-responders. Mortality, possibly related to radioembolisation, was low (4%) and similar to the expected mortality with surgical resection (median 3%) in patients with less advanced disease (Simmonds *et al*, 2006). Interestingly, a response to chemotherapy after radioembolisation was recorded in three patients; although this may have been, in part, a residual effect of the ongoing tumour shrinkage associated with radioembolisation that can be observed for several months after the initial inflammation and oedema (sometimes confused with disease progression) fade (Atassi *et al*, 2008).

In our study, the median survival of 12.6 months with ^90^Y resin microspheres is consistent with findings from previous retrospective analyses of similar metastatic CRC patients treated with ^90^Y microsphere radioembolisation. In these studies, median survival was 10.5 months for responding patients (compared to 4.5 months in non-responders) in one study (*n*=208 patients; Kennedy *et al*, 2006) and 10.5 months overall in the whole cohort in another study (*n*=41 patients; Jakobs *et al*, 2008). In these studies and in our trial, radioembolisation was only evaluated in patients in whom currently available therapies had failed and who were ineligible for liver-directed therapy including radiofrequency ablation, intensity-modulated radiotherapy or stereotactic radiotherapy.

The results of our trial evaluating radioembolisation beyond 3 or 4 lines of chemotherapy match favourably with other trials of systemic chemotherapy. In this treatment setting, median survivals are 4.5 months for historical controls (Kennedy *et al*, 2006), and 6.4–10.0 months with irinotecan (Fuchs *et al*, 2003; Schoemaker *et al*, 2004; Van Cutsem *et al*, 2005; Seymour *et al*, 2007; Sobrero *et al*, 2008), 6.3–9.3 months for panitumumab (Van Cutsem *et al*, 2007, 2008), 8.6–10.7 months for irinotecan/cetuximab (Cunningham *et al*, 2004; Sobrero *et al*, 2008; Wilke *et al*, 2008), 10.8 months for FOLFOX4, 10.2 months for bevacizumab and 9.5–12.9 months for FOLFOX4 or FOLFIRI and bevacizumab combined (Giantonio *et al*, 2007; Kang *et al*, 2009) as second, third or subsequent lines of systemic therapy in phase II/III studies. Recently, two small studies from the Paul Brousse Hospital in Paris have shown median survivals of 18.0 and 13.7 months with chronomodulated hepatic arterial infusion chemotherapy, and systemic circadian chronomodulated chemotherapy plus cetuximab, as a second or subsequent line of therapy (Bouchahda *et al*, 2009; Lévi *et al*, 2010). However, the greater part of the evidence would seem to indicate that locoregional chemotherapy appears to provide little or no additional benefit compared with systemic chemotherapy for the management of unresectable colorectal liver metastases (Mocellin *et al*, 2009; Pilati *et al*, 2009); although their combined benefit appears to merit further research (Alberts *et al*, 2010; Goéré *et al*, 2010).

The positive outcome of our trial inevitably raises questions about whether the selection of patients with disease limited to the liver, who had survived following multiple lines of therapy, had benefited from previous therapy rather than radioembolisation. To answer this question, it has been shown in a recent evaluation that radioembolisation, compared with best supportive care in a contemporary treatment setting, was the most significant predictor of progression-free survival (*P*=0.003) and survival (*P*<0.001) for chemorefractory CRC liver disease in a multivariate Cox proportional hazard model (Ricke *et al*, 2009). Remarkably, two patients from our trial were sufficiently downsized to enable potentially curative resection of ⩾3 hepatic segments. This is consistent with the experience from other centres where radioembolisation of similar patients has converted unresectable to resectable disease and resulted in prolonged survival (Van den Eynde *et al*, 2008).

Earlier treatment with ^90^Y resin microspheres in patients with unresectable CRC liver metastases is likely to improve the efficacy and safety of radioembolisation as post-chemotherapy damage to the normal hepatic parenchyma may increase the risk of treatment-related toxicity such as radiation-induced liver disease (Sangro *et al*, 2008). As the course of mCRC progresses, more patients are likely to be excluded from treatment using radioembolisation due to the development of extra-hepatic metastases, excessive hepatic tumour burden and/or compromised residual liver function. Therefore, using radioembolisation at an earlier point in the treatment of advanced disease may enable a greater proportion of patients to benefit from this therapy and provides the opportunity to combine this approach with suitable radio-sensitising chemotherapy regimens.

Pelvic radiotherapy combined with chemotherapy is already the gold standard for neo-adjuvant and adjuvant therapies of primary rectal cancer (Deutsch *et al*, 2007). Thus, CRC is known to be responsive to chemoradiotherapy in a significant portion of patients. The addition of radioembolisation to first-line 5-FU/LV systemic chemotherapy has already been shown in a small randomised controlled trial to provide a significant increase in overall survival and time to progression of disease for patients with unresectable mCRC, compared with 5-FU/LV systemic chemotherapy alone (29.4 *vs* 12.8 months, *P*=0.025) (van Hazel *et al*, 2004). Moreover, the overall responses rates were higher (72.7 *vs* 24.0%) and the time to progression was longer (18.6 *vs* 3.7 months) when radioembolisation was combined with systemic chemotherapy as first-line therapy than when radioembolisation alone was used later in our patients as a salvage therapy (van Hazel *et al*, 2004).

In conclusion, the treatment response observed in this single-arm, phase II trial suggests that patients with liver-only or liver-dominant CRC metastases who are chemotherapy refractory and who remain fit should be considered for salvage therapy using radioembolisation. The results of this trial warrant the further investigation of radioembolisation in combination with an appropriate radio-sensitising chemotherapy regimen earlier in the course of the disease to maximise the clinical benefits for patients with liver-only or liver-predominant mCRC.

## Figures and Tables

**Table 1:** Baseline characteristics in 50 patients with unresectable, chemotherapy-refractory CRC liver metastases treated with ^90^Y hepatic artery radioembolisation

**Characteristic**	
*Age (years): mean; median (range)*	64; 67 (34–85)
	
*Sex:* n (%)	
Male	37 (74)
Female	13 (26)
	
*Primary tumour site:* n (%)	
Colon	41 (82)
Rectum	9 (18)
	
Interval from mCRC diagnosis to radioembolisation (months): mean; median (range)	19; 17 (6–71)
	
*WHO performance status*	
Median (range)	0 (0–3)
0	35 (70)
1	14 (28)
2	0 (0)
3	1 (2)
	
*Haemopoietic function, medians*	
Haemoglobin, g per 100 ml	11.40
Leucocytes, × 10^3^ per mm^3^	3.95
Neutrophils (%)	66.30%
Platelets, × 10^3^ per mm^3^	285
Total bilirubin, mg per 100 ml	0.92
Albumin, g per 100 ml	3.80
ALT, U l^−1^	55
AST, U l^−1^	58
ALP, U l^−1^	486
INR	1.06
	
*Prior resection:* n (%)	
Extra-hepatic	11 (22)
Hepatic	12 (24)
	
*Prior chemotherapy lines:* n (%)	
Three	12 (24)
Four	25 (50)
Five	13 (26)
Prior bevacizumab: *n* (%)	11 (22)
Prior cetuximab: *n* (%)	5 (10)
	
*Liver involvement:* n (%)	
<25%	20 (40)
25–50%	30 (60)
	
*Number of metastases:* n (%)	
⩽4	21 (42)
>4	29 (58)
Bilobar/unilobar: *n* (%)	35/15 (70/30)
Synchronous/metachronous: *n* (%)	36/14 (72/28)
Median size of metastases: mm (range)	50 (8–120)

Abbreviations: ALP=Alkaline phosphatase; ALT=Alanine transaminase; AST=Aspartate transaminase; INR=Ratio of prothrombin time to normal; mCRC=metastatic colorectal cancer; WHO=World Health Organization.

**Table 2:** Treatment-related morbidity following a single intra-arterial injection of ^90^Y resin microspheres

**Event and Number (%) patients**					
**Early**		**Intermediate**		**Late**	
**0–48 h**		**Days 3–30**		**Months 2 and 3**	
Fever	4	Fever	3	GI ulcers	2
Pain	3	Chronic pain	5		
Leucocytosis	1	Jaundice/Nausea/Fatigue	1		
Total	8 (16%)	Total	11 (22%)	Total	2 (4%)

Abbreviation: GI=gastrointestinal.

All events were WHO Grade 1 or 2.

## References

[bib1] A’Hern RP (2001) Sample size tables for exact single-stage phase II designs. Stat Med 20: 859–866 11252008 10.1002/sim.721

[bib2] Alberts SR, Roh MS, Mahoney MR, O’Connell MJ, Nagorney DM, Wagman L, Smyrk TC, Weiland TL, Lai LL, Schwarz RE, Molina R, Dentchev T, Bolton JS (2010) Alternating systemic and hepatic artery infusion therapy for resected liver metastases from colorectal cancer: a North Central Cancer Treatment Group (NCCTG)/National Surgical Adjuvant Breast and Bowel Project (NSABP) phase II intergroup trial, N9945/CI-66. J Clin Oncol 28: 853–858 20048179 10.1200/JCO.2009.24.6728 PMC2834397

[bib3] Atassi B, Bangash AK, Bahrani A, Pizzi G, Lewandowski RJ, Ryu RK, Sato KT, Gates VL, Mulcahy MF, Kulik L, Miller F, Yaghmai V, Murthy R, Larson A, Omary RA, Salem R (2008) Multimodality imaging following ^90^Y radioembolization: a comprehensive review and pictorial essay. Radiographics 28: 81–99 18203932 10.1148/rg.281065721

[bib4] Bipat S, van Leeuwen MS, Ijzermans JN, Comans EF, Planting AS, Bossuyt PM, Greve JW, Stoker J (2007) Evidence-base guideline on management of colorectal liver metastases in the Netherlands. Neth J Med 65: 5–14 17293634

[bib5] Bouchahda M, Adam R, Giacchetti S, Castaing D, Brezault-Bonnet C, Hauteville D, Innominato PF, Focan C, Machover D, Lévi F (2009) Rescue chemotherapy using multidrug chronomodulated hepatic arterial infusion for patients with heavily pretreated metastatic colorectal cancer. Cancer 115: 4990–4999 19637365 10.1002/cncr.24549

[bib6] Cunningham D, Humblet Y, Siena S, Khayat D, Bleiberg H, Santoro A, Bets D, Mueser M, Harstrick A, Verslype C, Chau I, Van Cutsem E (2004) Cetuximab monotherapy and cetuximab plus irinotecan in irinotecan-refractory metastatic colorectal cancer. N Engl J Med 351: 337–345 15269313 10.1056/NEJMoa033025

[bib7] Deutsch E, Ezra P, Mangoni M, Ducreux M (2007) Radiotherapy for localized rectal cancer. Ann Oncol 18(Suppl 9): ix105–ix113 17631562 10.1093/annonc/mdm304

[bib8] Falcone A, Ricci S, Brunetti I, Pfanner E, Allegrini G, Barbara C, Crinò L, Benedetti G, Evangelista W, Fanchini L, Cortesi E, Picone V, Vitello S, Chiara S, Granetto C, Porcile G, Fioretto L, Orlandini C, Andreuccetti M, Masi G (2007) Phase III trial of infusional fluorouracil, leucovorin, oxaliplatin, and irinotecan (FOLFOXIRI) compared with infusional fluorouracil, leucovorin, and irinotecan (FOLFIRI) as first-line treatment for metastatic colorectal cancer: the Gruppo Oncologico Nord Ovest. J Clin Oncol 25: 1670–1676 17470860 10.1200/JCO.2006.09.0928

[bib9] Ferlay J, Autier P, Boniol M, Heanue M, Colombet M, Boyle P (2006) Estimates of the cancer incidence and mortality in Europe in 2006. Ann Oncol 18: 581–592 10.1093/annonc/mdl498 17287242

[bib10] Fuchs CS, Moore MR, Harker G, Villa L, Rinaldi D, Hecht JR (2003) Phase III comparison of two irinotecan dosing regimens in second-line therapy of metastatic colorectal cancer. J Clin Oncol 21: 807–814 12610178 10.1200/JCO.2003.08.058

[bib11] Gray B, Van Hazel G, Hope M, Burton M, Moroz P, Anderson J, Gebski V (2001) Randomised trial of SIR-Spheres plus chemotherapy vs. chemotherapy alone for treating patients with liver metastases from primary large bowel cancer. Ann Oncol 12: 1711–1720 11843249 10.1023/a:1013569329846

[bib12] Giantonio BJ, Catalano PJ, Meropol NJ, O’Dwyer PJ, Mitchell EP, Alberts SR, Schwartz MA, Benson III AB, Eastern Cooperative Oncology Group Study E3200 (2007) Bevacizumab in combination with oxaliplatin, fluorouracil, and leucovorin (FOLFOX4) for previously treated metastatic colorectal cancer: results from the Eastern Cooperative Oncology Group Study E3200. Clin Oncol 25: 1539–1544 10.1200/JCO.2006.09.6305 17442997

[bib13] Goéré D, Deshaies I, de Baere T, Boige V, Malka D, Dumont F, Dromain C, Ducreux M, Elias D (2010) Prolonged survival of initially unresectable hepatic colorectal cancer patients treated with hepatic arterial infusion of oxaliplatin followed by radical surgery of metastases. Ann Surg 251: 686–691 20224373 10.1097/SLA.0b013e3181d35983

[bib14] Hurwitz H, Fehrenbacher L, Novotny W, Cartwright T, Hainsworth J, Heim W, Berlin J, Baron A, Griffing S, Holmgren E, Ferrara N, Fyfe G, Rogers B, Ross R, Kabbinavar F (2004) Bevacizumab plus irinotecan, fluorouracil, and leucovorin for metastatic colorectal cancer. N Engl J Med 350: 2335–2342 15175435 10.1056/NEJMoa032691

[bib15] Jakobs TF, Hoffmann RT, Dehm K, Trumm C, Stemmler HJ, Tatsch K, La Fougere C, Murthy R, Helmberger TK, Reiser MF (2008) Hepatic yttrium-90 radioembolization of chemotherapy-refractory colorectal cancer liver metastases. J Vasc Interv Radiol 19: 1187–1195 18656012 10.1016/j.jvir.2008.05.013

[bib16] Kang BW, Kim TW, Lee JL, Ryu MH, Chang HM, Yu CS, Kim JC, Kim JH, Kang YK, Lee JS (2009) Bevacizumab plus FOLFIRI or FOLFOX as third-line or later treatment in patients with metastatic colorectal cancer after failure of 5-fluorouracil, irinotecan, and oxaliplatin: a retrospective analysis. Med Oncol 26: 32–37 18498064 10.1007/s12032-008-9077-8

[bib17] Kennedy AS, Coldwell D, Nutting C, Murthy R, Wertman Jr DE, Loehr SP, Overton C, Meranze S, Niedzwiecki J, Sailer S (2006) Resin ^90^Y-microsphere brachytherapy for unresectable colorectal liver metastases: modern USA experience. Int J Radiat Oncol Biol Phys 65: 412–425 16690429 10.1016/j.ijrobp.2005.12.051

[bib18] Kennedy AS, Nutting C, Coldwell D, Gaiser J, Drachenberg C (2004) Pathologic response and microdosimetry of ^90^Y microspheres in man: review of four explanted whole livers. Int J Radiat Oncol Biol Phys 60: 1552–1563 15590187 10.1016/j.ijrobp.2004.09.004

[bib19] Khatri VP, Chee KG, Petrelli NJ (2007) Modern multimodality approach to hepatic colorectal metastases: solutions and controversies. Surg Oncol 16: 71–83 17532622 10.1016/j.suronc.2007.05.001

[bib20] Lévi F, Karaboué A, Gorden L, Innominato PF, Saffroy R, Giacchetti S, Hauteville D, Guettier C, Adam R, Bouchahda M (2010) Cetuximab and circadian chronomodulated chemotherapy as salvage treatment for metastatic colorectal cancer (mCRC): safety, efficacy and improved secondary surgical resectability. Cancer Chemother Pharmacol (e-pub ahead of print) 10.1007/s00280-010-1327-8 20401611

[bib21] Manfredi S, Lepage C, Hatem C, Coatmeur O, Faivre J, Bouvier AM (2006) Epidemiology and management of liver metastases from colorectal cancer. Ann Surg 244: 254–259 16858188 10.1097/01.sla.0000217629.94941.cf PMC1602156

[bib22] McMillan DC, McArdle CS (2007) Epidemiology of colorectal liver metastases. Surg Oncol 16: 3–5 17493802 10.1016/j.suronc.2007.04.008

[bib23] Mocellin S, Pasquali S, Nitti D (2009) Fluoropyrimidine-HAI (hepatic arterial infusion) versus systemic chemotherapy (SCT) for unresectable liver metastases from colorectal cancer. Cochrane Database Syst Rev 8(3): CD007823 10.1002/14651858.CD007823.pub2 PMC13061358 19588444

[bib24] Nordlinger B, Sorbye H, Glimelius B, Poston GJ, Schlag PM, Rougier P, Bechstein WO, Primrose JN, Walpole ET, Finch-Jones M, Jaeck D, Mirza D, Parks RW, Collette L, Praet M, Bethe U, Van Cutsem E, Scheithauer W, Gruenberger T (2008) Perioperative chemotherapy with FOLFOX4 and surgery versus surgery alone for resectable liver metastases from colorectal cancer (EORTC intergroup trial 40983): a randomised controlled trial. Lancet 371: 1007–1016 18358928 10.1016/S0140-6736(08)60455-9 PMC2277487

[bib25] Pilati P, Mammano E, Mocellin S, Tessari E, Lise M, Nitti D (2009) Hepatic arterial infusion for unresectable colorectal liver metastases combined or not with systemic chemotherapy. Anticancer Res 29: 4139–4144 19846962

[bib26] Poston GJ (2004) Surgical strategies for colorectal liver metastases. Surg Oncol 13: 125–136 15572095 10.1016/j.suronc.2004.08.001

[bib27] Ricke J, Rühl R, Seidensticker M, Dudeck O, Pech M, Amthauer H (2009) Safety and efficacy of ^90^Y microsphere therapy in patients with extensive liver-dominant colorectal (CRC) metastases failing multiple lines of systemic chemotherapy: a matched-pair analysis. WCGIC Ann Oncol 20(Suppl 6): PD-002 (abstr)

[bib28] Sangro B, Gil-Alzugaray B, Rodriguez J, Sola I, Martinez-Cuesta A, Viudez A, Chopitea A, Iñarrairaegui M, Arbizu J, Bilbao JI (2008) Liver disease induced by radioembolization of liver tumors. Cancer 112: 1538–1546 18260156 10.1002/cncr.23339

[bib29] Schoemaker N, Kuppens I, Moiseyenko V, Glimelius B, Kjaer M, Starkhammer H, Richel DJ, Smaaland R, Bertelsen K, Poulsen JP, Voznyi E, Norum J, Fennelly D, Tveit KM, Garin A, Gruia G, Mourier A, Sibaud D, Lefebvre P, Beijnen JH, Schellens JH, ten Bokkel Huinink WW (2004) A Randomised phase II multicentre trial of irinotecan (CPT-11) using four different schedules in patients with metastatic colorectal cancer. Br J Cancer 91: 1434–1441 15381932 10.1038/sj.bjc.6602172 PMC2409929

[bib30] Seymour MT, Maughan TS, Ledermann JA, Topham C, James R, Gwyther SJ, Smith DB, Shepherd S, Maraveyas A, Ferry DR, Meade AM, Thompson L, Griffiths GO, Parmar MK, Stephens RJ (2007) Different strategies of sequential and combination chemotherapy for patients with poor prognosis advanced colorectal cancer (MRC FOCUS): a randomised controlled trial. Lancet 370: 143–152 17630037 10.1016/S0140-6736(07)61087-3

[bib31] Simmonds PC, Primrose JN, Colquitt JL, Garden OJ, Poston GJ, Rees M (2006) Surgical resection of hepatic metastases from colorectal cancer: a systematic review of published studies. Br J Cancer 94: 982–999 16538219 10.1038/sj.bjc.6603033 PMC2361241

[bib32] Sobrero AF, Maurel J, Fehrenbacher L, Scheithauer W, Abubakr YA, Lutz MP, Vega-Villegas ME, Eng C, Steinhauer EU, Prausova J, Lenz HJ, Borg C, Middleton G, Kröning H, Luppi G, Kisker O, Zubel A, Langer C, Kopit J, Burris III HA (2008) EPIC: phase III trial of cetuximab plus irinotecan after fluoropyrimidine and oxaliplatin failure in patients with metastatic colorectal cancer. J Clin Oncol 26: 2311–2319 18390971 10.1200/JCO.2007.13.1193

[bib33] Van Cutsem E, Dirix L, Van Laethem J, Van Belle S, Borner M, Gonzalez Baron M, Roth A, Morant R, Joosens E, Gruia G, Sibaud D, Bleiberg H (2005) Optimisation of irinotecan dose in the treatment of patients with metastatic colorectal cancer after 5-FU failure: results from a multinational, randomised phase II study. Br J Cancer 92: 1055–1062 15756271 10.1038/sj.bjc.6602462 PMC2361950

[bib34] Van Cutsem E, Peeters M, Siena S, Humblet Y, Hendlisz A, Neyns B, Canon JL, Van Laethem J-L, Maurel J, Richardson G, Wolf M, Amado RG (2007) Open-label phase III trial of panitumumab plus best supportive care compared with best supportive care alone in patients with chemotherapy-refractory metastatic colorectal cancer. J Clin Oncol 25: 1658–1664 17470858 10.1200/JCO.2006.08.1620

[bib35] Van Cutsem E, Siena S, Humblet Y, Canon JL, Maurel J, Bajetta E, Neyns B, Kotasek D, Santoro A, Scheithauer W, Spadafora S, Amado RG, Hogan N, Peeters M (2008) An open-label, single-arm study assessing safety and efficacy of panitumumab in patients with metastatic colorectal cancer refractory to standard chemotherapy. Ann Oncol 19: 92–98 17785764 10.1093/annonc/mdm399

[bib36] Van den Eynde M, Flamen P, El Nakadi I, Liberale G, Delatte P, Larsimont D, Hendlisz A (2008) Inducing resectability of chemotherapy refractory colorectal liver metastasis by radioembolization with yttrium-90 microspheres. Clin Nucl Med 33: 697–699 18806572 10.1097/RLU.0b013e318184b9a0

[bib37] van Hazel G, Blackwell A, Anderson J, Price D, Moroz P, Bower G, Cardaci G, Gray B (2004) Randomised phase 2 trial of SIR-spheres plus fluorouracil/leucovorin chemotherapy versus fluorouracil/leucovorin chemotherapy alone in advanced colorectal cancer. J Surg Oncol 88: 78–85 15499601 10.1002/jso.20141

[bib38] Wilke H, Glynne-Jones R, Thaler J, Adenis A, Preusser P, Aguilar EA, Aapro MS, Esser R, Loos AH, Siena S (2008) Cetuximab plus irinotecan in heavily pretreated metastatic colorectal cancer progressing on irinotecan: MABEL study. J Clin Oncol 26: 5335–5343 18854570 10.1200/JCO.2008.16.3758

